# Monthly At-Home Computerized Cognitive Testing to Detect Diminished Practice Effects in Preclinical Alzheimer's Disease

**DOI:** 10.3389/fnagi.2021.800126

**Published:** 2022-01-13

**Authors:** Roos J. Jutten, Dorene M. Rentz, Jessie F. Fu, Danielle V. Mayblyum, Rebecca E. Amariglio, Rachel F. Buckley, Michael J. Properzi, Paul Maruff, Craig E. Stark, Michael A. Yassa, Keith A. Johnson, Reisa A. Sperling, Kathryn V. Papp

**Affiliations:** ^1^Department of Neurology, Massachusetts General Hospital and Harvard Medical School, Boston, MA, United States; ^2^Department of Neurology, Brigham and Women's Hospital and Harvard Medical School, Boston, MA, United States; ^3^Department of Radiology, Massachusetts General Hospital and Harvard Medical School, Boston, MA, United States; ^4^Melbourne School of Psychological Sciences, University of Melbourne, Melbourne, VIC, Australia; ^5^CogState Ltd., Melbourne, VIC, Australia; ^6^The Florey Institute of Neuroscience and Mental Health, University of Melbourne, Melbourne, VIC, Australia; ^7^Department of Neurobiology and Behavior, Center for the Neurobiology of Learning and Memory, University of California, Irvine, Irvine, CA, United States

**Keywords:** computerized testing, remote assessment, practice effects, digital biomarkers, preclinical AD

## Abstract

**Introduction:** We investigated whether monthly assessments of a computerized cognitive composite (C3) could aid in the detection of differences in practice effects (PE) in clinically unimpaired (CU) older adults, and whether diminished PE were associated with Alzheimer's disease (AD) biomarkers and annual cognitive decline.

**Materials and Methods:** *N* = 114 CU participants (age 77.6 ± 5.0, 61% female, MMSE 29 ± 1.2) from the Harvard Aging Brain Study completed the self-administered C3 monthly, at-home, on an iPad for one year. At baseline, participants underwent in-clinic Preclinical Alzheimer's Cognitive Composite-5 (PACC5) testing, and a subsample (*n* = 72, age = 77.8 ± 4.9, 59% female, MMSE 29 ± 1.3) had 1-year follow-up in-clinic PACC5 testing available. Participants had undergone PIB-PET imaging (0.99 ± 1.6 years before at-home baseline) and Flortaucipir PET imaging (*n* = 105, 0.62 ± 1.1 years before at-home baseline). Linear mixed models were used to investigate change over months on the C3 adjusting for age, sex, and years of education, and to extract individual covariate-adjusted slopes over the first 3 months. We investigated the association of 3-month C3 slopes with global amyloid burden and tau deposition in eight predefined regions of interest, and conducted Receiver Operating Characteristic analyses to examine how accurately 3-month C3 slopes could identify individuals that showed >0.10 SD annual decline on the PACC-5.

**Results:** Overall, individuals improved on all C3 measures over 12 months (β = 0.23, 95% CI [0.21–0.25], *p* < 0.001), but improvement over the first 3 months was greatest (β = 0.68, 95% CI [0.59–0.77], *p* < 0.001), suggesting stronger PE over initial repeated exposures. However, lower PE over 3 months were associated with more global amyloid burden (*r* = −0.20, 95% CI [−0.38 – −0.01], *p* = 0.049) and tau deposition in the entorhinal cortex (*r* = −0.38, 95% CI [−0.54 – −0.19], *p* < 0.001) and inferior-temporal lobe (*r* = −0.23, 95% CI [−0.41 – −0.02], *p* = 0.03). 3-month C3 slopes exhibited good discriminative ability to identify PACC-5 decliners (AUC 0.91, 95% CI [0.84–0.98]), which was better than baseline C3 (*p* < 0.001) and baseline PACC-5 scores (*p* = 0.02).

**Conclusion:** While PE are commonly observed among CU adults, diminished PE over monthly cognitive testing are associated with greater AD biomarker burden and cognitive decline. Our findings imply that unsupervised computerized testing using monthly retest paradigms can provide rapid detection of diminished PE indicative of future cognitive decline in preclinical AD.

## Introduction

Alongside the increased focus on characterizing Alzheimer's disease (AD) in the preclinical stage, there is a need to detect and track the cognitive changes that may emerge during this stage more rapidly. However, capturing short-term cognitive changes in preclinical AD is a major challenge using conventional paper-and-pencil cognitive tests, which typically require in-clinic assessments at annual intervals and only detect subtle decline over multiple years (Petersen et al., 2016; Mortamais et al., 2017; Jutten et al., 2020b). This is a particular hurdle for AD secondary prevention trials, which currently require large sample-sizes and lengthy follow-up to enable the detection of an attenuation of subtle cognitive decline.

Computerized cognitive testing has the potential to capture changes in cognition earlier, by enabling standardized administration and data analyses allowing for remote, unsupervised, and more frequent assessments (e.g., monthly rather than yearly) in a feasible way (Gold et al., 2018; Koo and Vizer, 2019). Several computerized tests have been developed for use in remote, unsupervised settings, including the Computerized Cognitive Composite (C3) battery, which was designed to assess cognitive processes that rely on the medial temporal lobe (MTL) (Rentz et al., 2016; Buckley et al., 2017; Papp et al., 2021b). The C3 comprises two well-validated episodic memory paradigms: the Face Name Associative Memory Exam (FNAME) (Rentz et al., 2011) and the Behavioral Pattern Separation Task—Object Version (BPSO) (Stark et al., 2013), and the Cogstate Brief Battery (CBB) (Maruff et al., 2009; Lim et al., 2012). It was recently shown that unsupervised, at-home C3 testing on an iPad was feasible and could provide data that discriminated reliably between cognitively normal and impaired adults (Rentz et al., 2016; Buckley et al., 2017; Papp et al., 2021b).

The higher frequency assessments afforded through use of computerized tests enable the study of practice effects (PE) that can occur with repeated cognitive assessments (Beglinger et al., 2005). PE have typically been viewed as a source of bias (Salthouse, 2012), but several studies showed that characterizing PE could provide an indicator of cognitive impairment and, more specifically, that lower PE reflect a decreased ability to benefit from previous experience when re-exposed ot the same stimuli (Duff et al., 2007, 2012; Jutten et al., 2020a). PE have been reported for the individual C3 and CBB measures when administered in clinically unimpaired (CU) adults (Baker et al., 2019; Samaroo et al., 2020; Stricker et al., 2020). Interestingly, the study by Samaroo et al. revealed *diminished* PE on the FNAME test in CU with high levels of amyloid compared to CU with low levels of amyloid, which was evident from only 4 months of repeated assessments. This suggests that failure of learning due to practice may already be evident in preclinical AD, and that the magnitude of PE may have potential as a cognitive marker of this very early manifestation of the disease.

The current study expands on previous work by investigating whether characterizing PE across a range of memory tasks included in the C3 battery could aid in the detection of early cognitive change in preclinical AD. First, we investigated the nature and magnitude of PE that arose from monthly repeated exposure to at-home C3 assessments over 1 year. Upon seeing improvement, we investigated whether PE on computerized testing could be observed over the first 3 months, as we expected that the PE signal would be strongest over the first 4–5 assessments (Watson et al., 1994; Calamia et al., 2012; Samaroo et al., 2020). Next, we examined the relationship of individual variation in shorter term PE (i.e., 3 months) with (1) AD biomarker burden measured using neuroimaging and (2) cognitive decline on standard paper-pencil cognitive testing over 1 year (Petersen et al., 2021).

## Materials and Methods

### Study Participants

The current study describes data from the At-Home Digital Cognition Sub-Study including participants from the Harvard Aging Brain Study (HABS). HABS is an ongoing longitudinal observational cohort-study of community-dwelling older adults who are clinically normal at the time of enrollment. Inclusion criteria for HABS have been described in detail elsewhere (Dagley et al., 2017). The At-Home Digital Cognition Study started recruiting participants in the 6th HABS year. For participation in the At-Home Digital Cognition Study, participants were deemed to be CU at the start of the study, which was determined by clinician consensus based on cognitive and functional test results and medical history (Papp et al., 2020). The study was approved by an ethical review board, and all participants provided written informed consent.

### Measures

#### Computerized Cognitive Composite

The C3 battery is a self-administered test battery presented on an iPad using CogState software. It includes the Face Name Associative Memory Exam (FNAME) (Rentz et al., 2011), a version of the Behavioral Pattern Separation Task-Object version (BPSO) (Stark et al., 2013), and the Cogstate Brief Battery comprising four brief tests: the Detection Task (DET), the Identification Task (IDN), One Card Learning Task (OCL) and the One-Back Task (ONB) (Maruff et al., 2009). The C3 and its individual measures have been described in detail elsewhere (Papp et al., 2021b), and Supplementary Table 1 provides a detailed overview of the individual outcomes.

Briefly, the FNAME is an associative memory paradigm requiring participants to encode and subsequently recall and match faces with corresponding names. Participants are shown 12 face-name pairs, and after a 12–15-min delay there are three measures of memory including first letter name recall, face-name matching and face recognition. For the current study, we focused on the free recall measure, i.e., the first letter name recall test (FNLT), since this is the FNAME measure that is expected to have the fewest range restrictions in scoring and therefore most likely to capture PE over repeated exposures. Participants are asked to select the first letter of the name paired with that face, and the primary outcome is the number of first letters correctly recalled. Total score range is 0–12 with higher scores reflecting better performance.

For the BPSO, participants are presented with a series of unique images (encoding phase) and encouraged to attend carefully to the physical characteristics of each object by having them decide whether the object is used mostly outdoors or indoors. This is followed by a recognition phase that includes repeated, novel and distractor images (lures), which participants are asked to categorize into Old, Similar, or New. Of the images presented during the recognition phase, one third are identical to those presented during encoding (for which the correct response would be “Old”), one third of the images contains an object that is visually similar, but not identical to an object presented during the encoding phase (i.e., lures, for which correct response would be “Similar”) and one third are objects that had not been seen during encoding (i.e., foils, for which the correct response would be “New”). The version of the BPSO that was used in the current study differs from the original version in that the studied items brought into the recognition phase are presented both as repeated identical targets and as similar lures, with half of the items having the target version presented first and half of the items having the similar lure version presented first. The primary outcome of the BPSO is a metric reflecting the ability to correctly discriminate between stimuli that are similar but not identical to previously learned items. That is, a Lure Discrimination Index (LDI) is calculated as: Proportion of “similar” responses made to Lure trials minus the proportion of “similar” responses to Foil trials. The LDI range is 0–1, with higher scores reflecting better performance.

The CBB uses playing cards as stimuli to measure reaction time and working memory. The DET is a measure of attention, and participants are asked to respond when a stimulus card is turned face up. The IDN is a measure of attention and inhibitory control, where a respondent must choose whether a flipped card is red or not. Primary outcome measures for the DET and IDN are reaction time. The OCL task is a non-verbal continuous memory task in which playing cards are shown one at a time with a subset of the cards repeating several times throughout the task. The ONB task measures working memory by requiring participants to serially match each card to the previous trial. Outcome measures for the OCL and ONB include both reaction time and number of correct responses.

#### In-clinic Cognitive Testing

Participants underwent standard paper-and-pencil in-clinic cognitive testing including the Preclinical Alzheimer's Cognitive Composite 5 (PACC-5) (Donohue et al., 2014; Papp et al., 2017). The PACC5 is a widely used cognitive outcome measure in research and clinical trials of preclinical AD and comprises well-validated paper-and-pencil tests including the Mini-Mental State Examination (MMSE) (Folstein et al., 1975), the Wechsler Memory Scale-Revised Logical Memory Delayed Recall (Wechsler, 1987), the Digit-Symbol Coding Test (Wechsler, 2008), the Free and Cued Selective Reminding Test Free + Total Recall (Grober et al., 2009), and the Category Fluency Test (Monsch et al., 1992). Here, the PACC5 is computed as an averaged z-score of all individual measures.

#### Amyloid and Tau Biomarkers

We used neuro imaging to investigate whether the magnitude of PE was associated with global amyloid burden and regional tau deposition, since our current understanding of preclinical AD is that amyloid pathology is diffusely distributed across brain areas (Villemagne et al., 2011; Mormino et al., 2014) whereas tau deposition is initially focally present in the MTL regions (Johnson et al., 2016; Hanseeuw et al., 2019) where it is found to be associated with episodic performance (Maass et al., 2018). Amyloid burden and tau deposition were measured and quantified using positron-emission tomography (PET) imaging using ^11^C-Pittsburg Compound-B (PiB) and ^18^F-Flortaucipir (FTP), respectively, in accordance with established protocols for acquisition and analysis (Mormino et al., 2014; Johnson et al., 2016). Briefly, PiB images were acquired using a 60-min dynamic acquisition and FTP images were acquired from 75 to 105 min post-injection on a Siemens ECAT HR+ PET scanner. Following acquisition, a mean PET image was created and coregistered with the corresponding T1 MR image using the SPM12 package (Wellcome Centre for Human Neuroimaing) and the resulting coregistration transformation matrices were saved. FreeSurfer (v6) regions of interest (ROIs) defined by segmenting the MR images were transformed into the PET native space using the inverse transformation matrices. PiB was expressed as the distribution volume ratio (DVR, estimated with reference Logan graphical method), and FTP as an averaged standardized uptake value ratio (SUVR) over 70–105 min corrected for partial volume effects (PVC). For both PiB and FTP, bilateral cerebellum gray matter was used as the reference region for DVR and SUVr estimates respectively. For PiB, a global cortical aggregate was calculated for each participant based on the average PiB DVR in frontal, lateral temporoparietal, and retrosplenial (FLR) regions, and participants were dichotomized into low (Aβ−) vs. high (Aβ+) groups (DVR cut-off-1.185). For FTP, we used the SUVR PVC values of eight predefined ROIs: the entorhinal cortex, inferior temporal lobe, amygdala, hippocampus [adjusted for choroid plexus (Lee et al., 2018)], parahippocampal region, fusiform gyrus, precuneus and posterior cingulate region.

### Procedures

Baseline and conclusion of the At-Home Digital Cognition Study coincided with participants' annual HABS in-clinic visits. At baseline (In Clinic Visit 1), participants completed an iPad/Cogstate one-on-one training session with a trained HABS rater and completed the first C3 assessment in the clinic. Participants were then provided a study iPad to complete the C3 at home. The first At-Home C3 assessment was done independently at-home 1 week later (hereafter referred to as visit 0.25). Thereafter, participants completed the monthly C3 for 12 At-Home sessions with 4-week intervals. The final C3 administration occurred in-clinic as part of the second annual HABS visit (In-Clinic Visit 2), leading to a maximum of 15 C3 sessions. Participants received reminder calls prior to their scheduled test dates and were encouraged to complete the C3 at the same time monthly.

The C3 battery has a total administration time of 25–30 min. On screen instructions are provided, but participants do not receive feedback upon completion of any of the individual tests nor monthly assessments. Previous work indicated good feasibility and usability in unsupervised settings after one in-clinic training session, with a high percentage of older individuals completed at-home assessments correctly including those with lower computer literacy (Rentz et al., 2016; Samaroo et al., 2020; Papp et al., 2021a).

The At-Home Digital Cognition study was initially designed with all C3 tests being repeated using alternating versions. However, a second version of the FNAME was added as well, repeating the same version, based on the hypothesis that repeating the same versions would lead to stronger PE. A recent study comparing monthly performance on the FNAME alternate vs. same versions confirmed this (Samaroo et al., 2020), and we therefore decided to focus on the FNAME same version in the current study. Thus, retest procedures differed across individual C3 measures investigated in the current study. For the FNAME, the same version was repeated each month (A-A-A-A). For the BPSO, four alternate versions were used following the same sequence for everyone (A-B-C-D). For the CBB measures, alternate versions were used each month and the sequence of versions was randomized for each participant.

### Statistical Analyses

Prior to statistical analyses, completion and performance checks were performed on all individual C3 measures to ensure the integrity of the data, by applying previously defined task-specific cut-offs (Supplementary Table 1). Scores that fell below these cut-offs were excluded from further analyses.

Statistical analyses were conducted in R version 4.0.3. Statistical significance was set at *p* < 0.05. To facilitate comparison across C3 measures, all data from all individual C3 measures were z-transformed using the overall sample mean and standard deviation (SD) at baseline. The BPSO, FNLT, and OCL accuracy z-scores were summed into an overall C3 z-score (Papp et al., 2021b). Linear mixed models (LMM) were used to investigate C3 performance over time (months, continuous) correcting for age, sex, and years of education. Since there were no significant interactions between time and covariates (i.e., age, sex, and years of education) for any of the C3 measures, interaction terms were not included in the final models. We initially ran the LMM including all follow-up data to describe monthly performance over 1 year, and subsequently repeated the same models including only follow-up data over the first 3 months to investigate the magnitude of PE over the initial assessments. Mean to standard deviation ratios (MSDRs) were calculated for each measure to compare effect-sizes across measures over 3 months. Figures showing the mean, SD and 95% confidence interval (CI) by study visit (i.e., time as categorical variable) are provided to visualize the overall trajectory of C3 performance.

Next, individual covariate-adjusted slopes were extracted from the aforementioned LMM to quantify PE over 3 months for each participant. Pearson's correlations were computed to investigate the association between 3-month C3 slopes and baseline amyloid burden (PiB DVR, continuous) as well as tau deposition in the entorhinal cortex and inferior-temporal lobe (SUVR, partial-volume corrected). After observing that correlations between C3 slopes and FTP uptake in the entorhinal and inferior temporal regions were significant, we sought to explore the relationship with a potential pattern of tau uptake in these and other regions which have shown early accumulation (Johnson et al., 2016). To that end, FTP data was analyzed using Partial Least Squares (PLS) analysis performed using MATLAB. PLS is a data reduction technique that produces predictive models when data are highly collinear, and hence it can be applied to imaging data as multivariate analysis method for identifying spatial patterns that are optimally associated with task performance (McIntosh et al., 1996). An additional advantage is that PLS analysis may be more robust to noise in the data than univariate analysis. Here, we used PLS analysis as a *post-hoc* hypothesis-driven method to complement the univariate correlational analyses. We explored associations between C3 baseline as well as C3 slope measures and spatial distributions of tau uptake across eight ROI: the entorhinal cortex, inferior temporal lobe, amygdala, hippocampus [adjusted for choroid plexus (Lee et al., 2018)], parahippocampal region, fusiform, precuneus and posterior cingulate. PLS analysis was used to decompose the input data (FTP data for the eight ROI: SUVR, all PVC) into components that are maximally correlated with C3 slopes using MATLAB build-in function “plsregress.” The number of components was predefined to seven, as seven components accounted for at least 95% of the total variance in the input data based on principal component analysis (PCA, MATLAB build-in function “pca”). Only the first PLS component resulted in significant correlations between PLS scores for FTP SUVR data and C3 slopes. This remained the same when using fewer components. Therefore, the first PLS component, representing the spatial patterns of tau uptake that most correlated with the C3 measures, was used for further interpretations. We first ran PLS analyses in the overall sample (corrected for the total PiB FLR load) separately for all C3 measures (baseline scores as well as slopes), and then repeated the analyses separately in the Aβ− and Aβ+ groups. To protect from Type I Error, a Bonferroni correction was conducted (adjusted *p*-value <0.005). PLS weights (representing the contribution of each ROI to the overall spatial pattern) were z-transformed, and regions with a z-score weights > 1 or < −1 were considered significant. Five-fold cross-validation was used to minimize mean square errors. Figures including the optimal spatial pattern as well as the correlation with the presentation of this pattern and C3 slopes are provided.

Finally, individual PACC5 slopes were extracted using LMM correcting for age, sex, and education for participants with baseline and 1-year follow-up PACC5 testing available (*n* = 72). Pearson's correlations were used to assess the association between 3-month slopes on the C3 and change on the PACC5 over 1 year. We then conducted Receiver Operating Characteristic (ROC) analyses to quantify how accurately C3 slopes could identify individuals who would show more than 0.10 SD decline on the PACC5 over 1 year, which has previously been suggested as a clinically meaningful cut-off for annual decline in amyloid positive cognitively normal individuals (Papp et al., 2020; Petersen et al., 2021).

## Results

### Sample Characteristics

Baseline characteristics of the total sample (*N* = 114) as well as subsample with 1-year in-clinic follow-up (FU) available (*n* = 72) are presented in Table 1. All participants had undergone PiB-PET imaging (0.99 ± 1.6 years before at-home baseline) and FTP PET was available for the majority (*n* = 105, 0.62 ± 1.1 years before at-home baseline). Overall, adherence was high with an average of 11.7 (SD = 3.2) FU C3 assessments, 96% of the participants having at least 3 completed FU assessments, 91% having at least 6 completed FU assessments and 75% having completed 12 or 13 FU assessments (Supplementary Figure 1). Within-testing session discontinuation rates were low (3% in total over all observations on all C3 measures across all visits). Documented reasons for non-completion mainly included technological issues or lack of time. For completed assessments, integrity checks of individual assessments were high, the criterion for performance validity was met at a 99.2% for the BPSO, 99.1% for the DET, 98.9% IDN, 99% for ONB, 98.4% OCL, 99.8% for the FNLT. Compared to the total sample, the subsample (*n* = 72) with 1 year in-clinic follow-up had completed more C3 assessments (*p* < 0.001) but did not differ regarding other baseline clinical and demographic characteristics (all *p* > 0.05).

**Table 1:** Baseline characteristics for the overall sample and subsample with in-clinic follow-up after 1 year.

	**Total sample** **(*N* = 114)**	**Sample with in-clinic follow-up** **(*n* = 72)**
FU C3 assessments, M (SD) [range]	11.7 (3.2), [2–15]	12.8 (1.8), [2–15]^*^
N month 0.25/1/2/3	101/104/106/104	64/68/70/69
Age, M (SD)	77.6 (5.0)	77.8 (4.9)
Female, n (%)	70 (67.3%)	42 (60%)
Years of Education, M (SD)	16.5 (2.7)	16.3 (2.8)
Global CDR, 0/0.50	105/9	66/6
MMSE score, M (SD)	29.1 (1.3)	29.2 (1.2)
PACC5 score, M (SD)	0.22 (0.76)	0.29 (0.73)
PiB-PET years since C3 baseline	−0.99 ± 1.6	−0.68 ± 1.7
Global cortical amyloid (DVR)	1.21 ± 0.23	1.22 ± 0.25
Aβ status	81 Aβ−/33 Aβ+	50 Aβ−/22 Aβ+
N	105	66
FTP-PET years since C3 baseline	−0.62 ± 1.1	−0.34 ± 1.2
FTP-PET ET Tau (SUVR, PVC)	1.38 ± 0.28	1.39 ± 0.27
FTP-PET IT Tau (SUVR, PVC)	1.50 ± 0.18	1.50 ± 0.16

*N.B*.

* *p < 0.001*.

*C3, Computerized Cognitive Composite; CDR, Clinical Dementia Rating scale; MMSE, Mini-Mental State Examination; PACC-5, Preclinical Alzheimer's Cognitive Composite-−5; PET, Positron-emission tomography; PiB, ^11^C-Pittsburg Compound-B; Aβ, Amyloid-beta; DVR, distribution volume ratio; FTP, ^18^F-Flortaucipir; SUVR, Standardized uptake value ratio; PVC, Partial volume corrected*.

### Change Over Time on Monthly C3 Assessments

**Figure 1:**
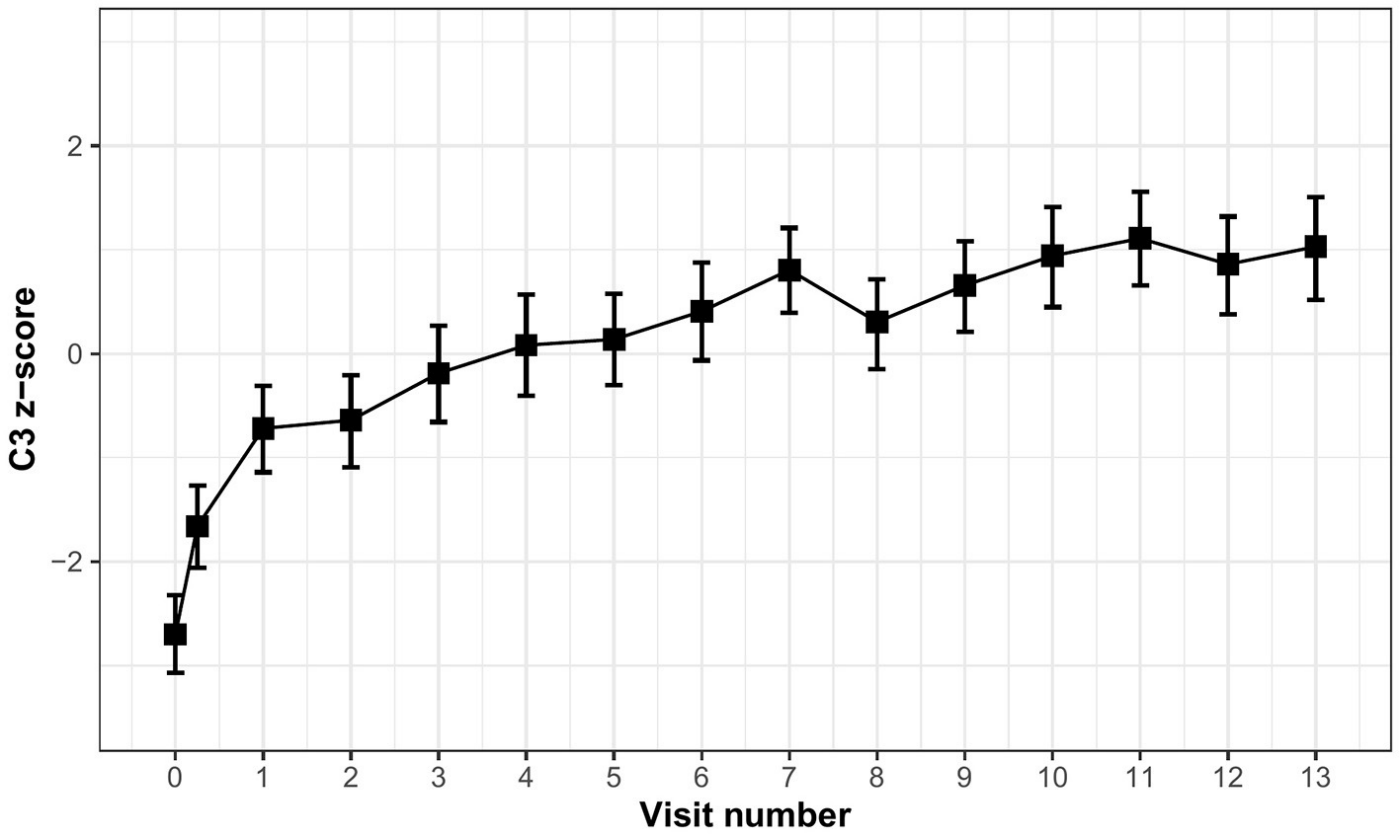
Mean trajectory of C3 performance over monthly visits.

Table 2 displays the time (in months) estimates obtained from LMM correcting for age, sex, and years of education for the C3 score as well as the individual C3 measures. Overall, individuals improved over 1 year on the C3 (β = 0.23, 95% CI [0.21–0.25], *p* < 0.001). However, improvement was greatest over the first 3 months (β = 0.68, 95% CI [0.59–0.77], *p* < 0.001) suggesting stronger practice over the initial exposures, which is also visualized by the mean trajectory of C3 performance over months (Figure 1).

**Table 2:** Time estimates extracted from linear mixed models corrected for age, sex, and education.

	**Monthly change over 1 year**			**Monthly change over first 3 months**			
	**Time**	**95% CI**	***P*-Value**	**Time**	**95% CI**	***P*-Value**	**MSDR**
C3	0.226	0.207–0.245	<0.001	0.678	0.587–0.768	<0.001	1.39
BPSO	0.073	0.061–0.085	<0.001	0.212	0.157–0.268	<0.001	0.71
FNLT	0.098	0.088–0.108	<0.001	0.379	0.328–0.429	<0.001	1.37
OCL acc	0.051	0.041–0.060	<0.001	0.072	0.020–0.125	0.007	0.25
ONB acc	0.034	0.023–0.044	<0.001	0.100	0.041–0.158	0.001	0.31
DET rt	−0.024	−0.036 to −0.012	<0.001	−0.051	−0.103–0.000	0.052	0.18
IDN rt	−0.009	−0.020–0.001	0.073	−0.062	−0.115 to −0.010	0.021	0.22
OCL rt	−0.032	−0.044 to −0.021	<0.001	−0.141	−0.188 to −0.095	<0.001	0.55
ONB rt	−0.052	−0.063 to −0.041	<0.001	−0.163	−0.207 to −0.118	<0.001	0.67

*N.B. C3 is summed z-score of BPSO + FNLT + OCL. Negative scores on reaction time measures reflect improvement*.

*C3, Computerized Cognitive Composite (computed as the sum of the BPSO, FNLT, and OCL accuracy z-scores); BPSO, Behavioral Pattern Separation Task—Object Version; FNLT, First Name Letter Test; OCL, One-Card Learning; ONB, One Back; DET, Detection; IDN, Identification; acc, accuracy; rt, reaction time; MSDR, mean to standard deviation ratio*.

When looking at the individual measures, a statistically significant improvement was observed on most individual measures over 1 year (all *p*-values <0.001), except for IDN reaction time (Table 2). Time estimates from the models including only the first 3 months were all greater than time estimates over 1 year, particularly for the BPSO and FNLT (Table 2). When comparing change over 3 months across the C3 measures, improvement was greater for the FNLT (MSDR 1.37) and BPSO (MSDR 0.71) as compared to the OCL and ONB accuracy measures (MSDRs 0.25 and 0.31 respectively). For both the OCL and ONB, the reaction time measures exhibited larger effect-sizes (MSDR 0.55 and 0.67 respectively) than the accuracy measures.

### Diminished Practice Over 3 Months Is Associated With AD Biomarker Burden

**Figure 2:**
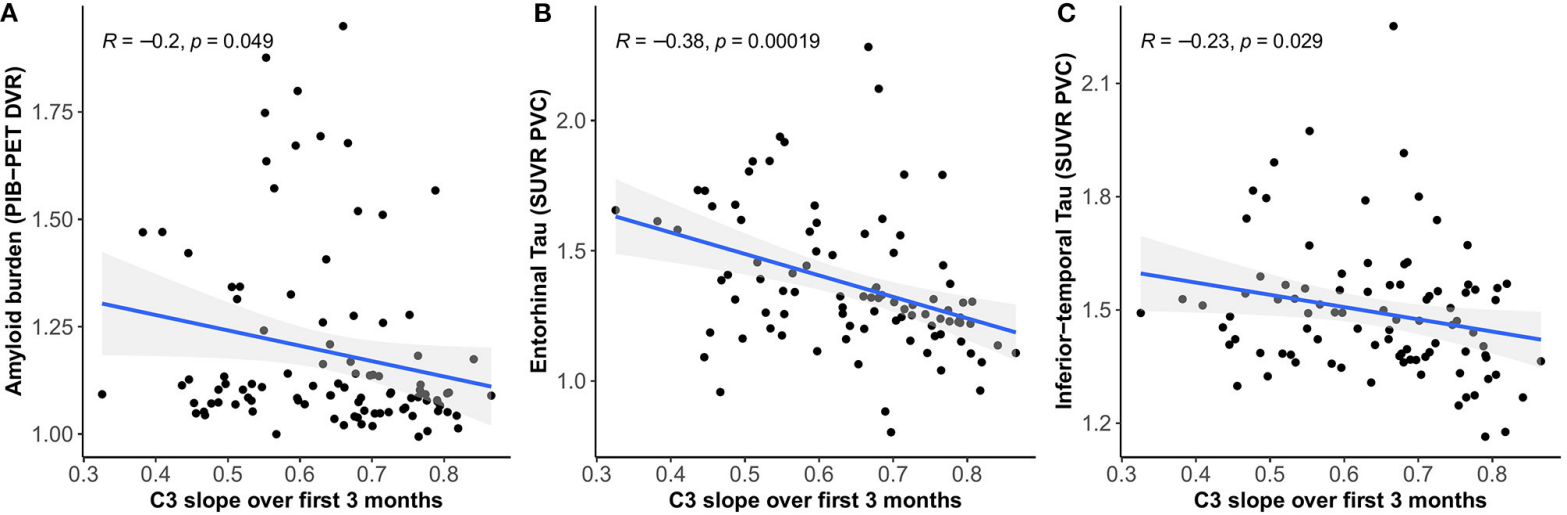
Relationship between C3 slopes over 3 months and global amyloid burden **(A)**, tau deposition in the entorhinal cortex **(B)**, and inferior-temporal lobe **(C)**.

We found moderate negative correlations between 3-month C3 slopes (covariate adjusted) and cross-sectional global amyloid burden (*r* = −0.20, 95% CI [−0.38 – −0.01], *p* = 0.049) (Figure 2A) as well as tau deposition in the entorhinal cortex (*r* = −0.38, 95% CI [−0.54 – −0.19], *p* < 0.001) (Figure 2B) and inferior-temporal lobe (*r* = −0.23, 95% CI [−0.41 – −0.02], *p* = 0.033) (Figure 2C), indicating that less improvement over 3 months is associated with greater amyloid and tau burden.

**Figure 3:**
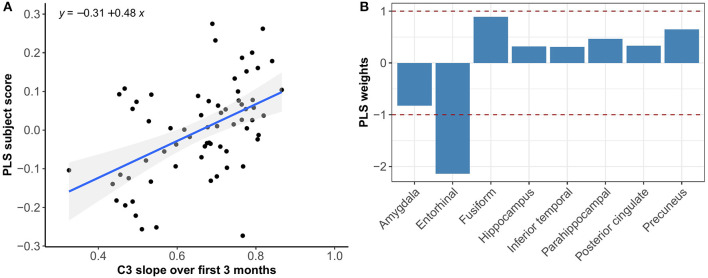
Association between optimal spatial distribution of tau and C3 slopes over 3 months in the Aβ− group. Left panel **(A)** shows the correlation between PLS subject scores (expression of spatial patterns of tau distribution) and C3 slopes; right panel **(B)** presents the spatial patterns of tau distribution of z-transformed PLS weights in each ROI.

**Figure 4:**
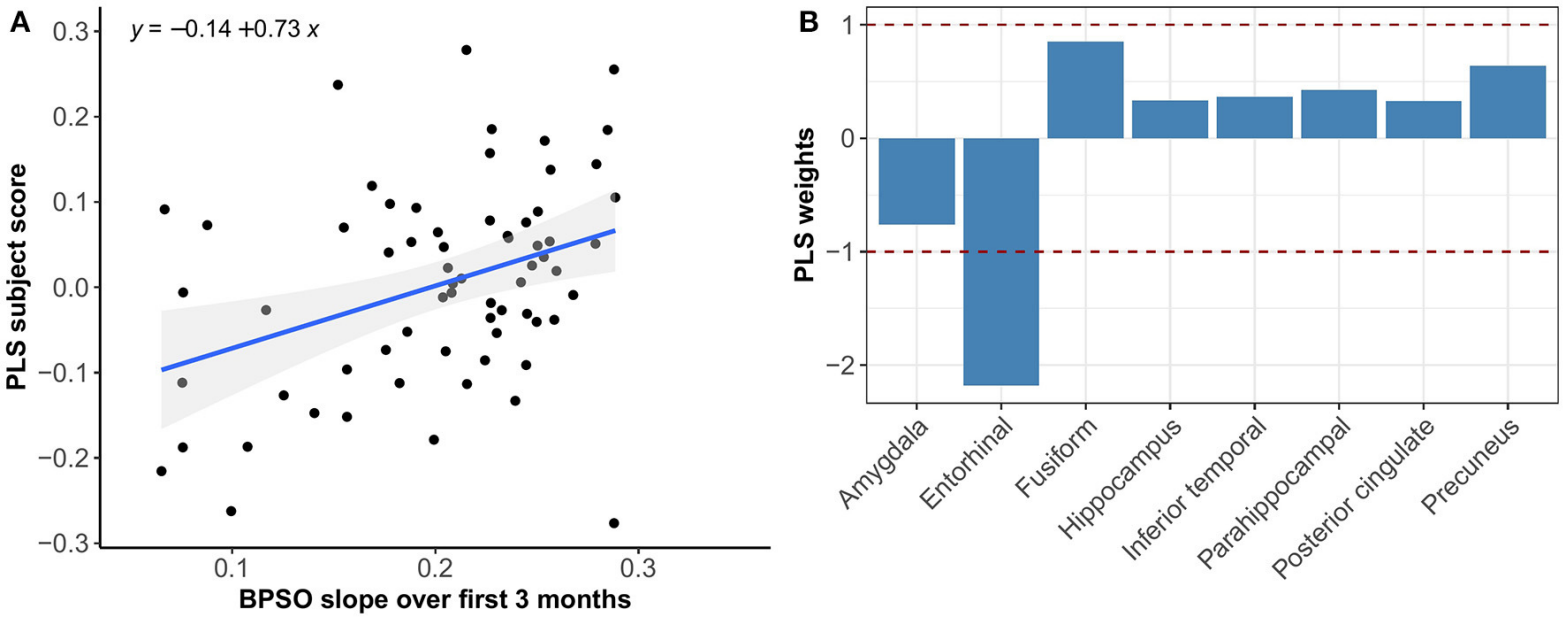
Association between optimal spatial distribution of tau and BPSO slopes over 3 months in the Aβ− group. Left panel **(A)** shows the correlation between PLS subject scores (expression of spatial patterns of tau distribution) and BPSO slopes; right panel **(B)** presents the spatial patterns of tau distribution of z-transformed PLS weights in each ROI.

**Figure 5:**
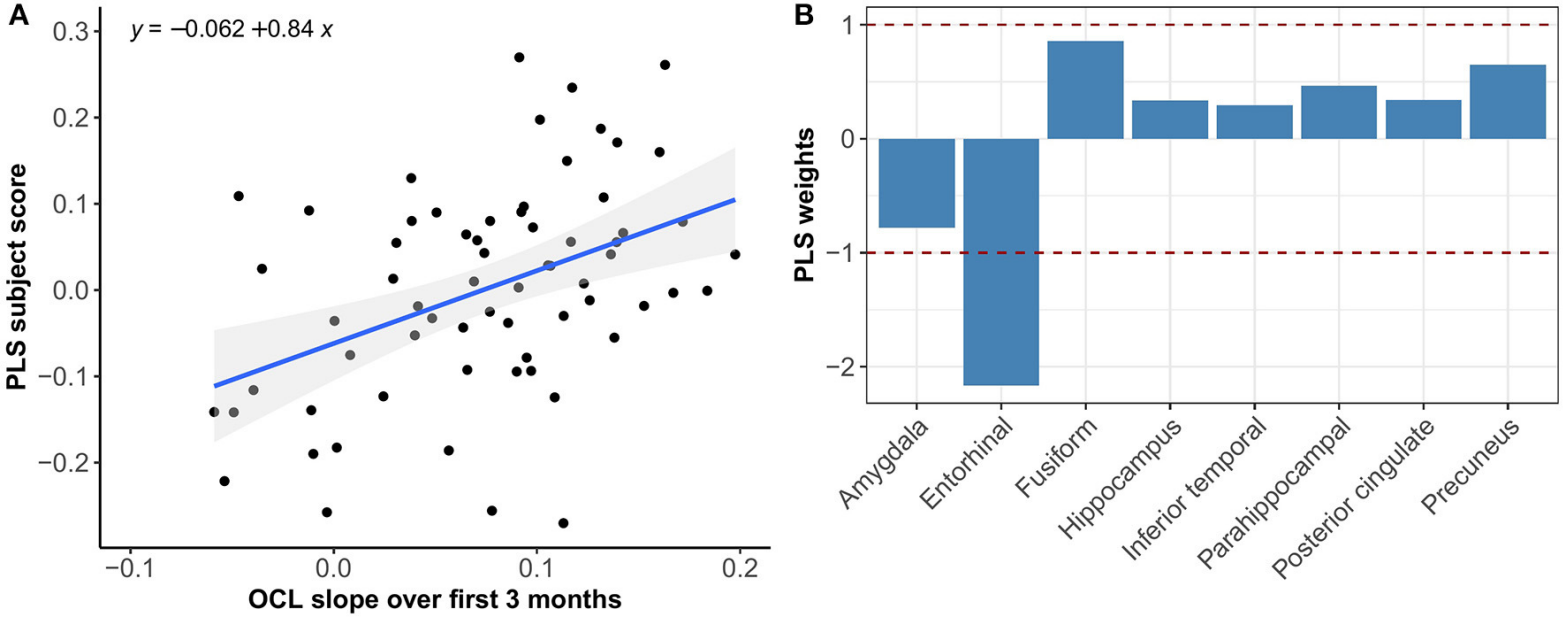
Association between optimal spatial distribution of tau and OCL slopes over 3 months in the Aβ− group. Left panel **(A)** shows the correlation between PLS subject scores (expression of spatial patterns of tau distribution) and OCL slopes; right panel **(B)** presents the spatial patterns of tau distribution of z-transformed PLS weights in each ROI.

**Figure 6:**
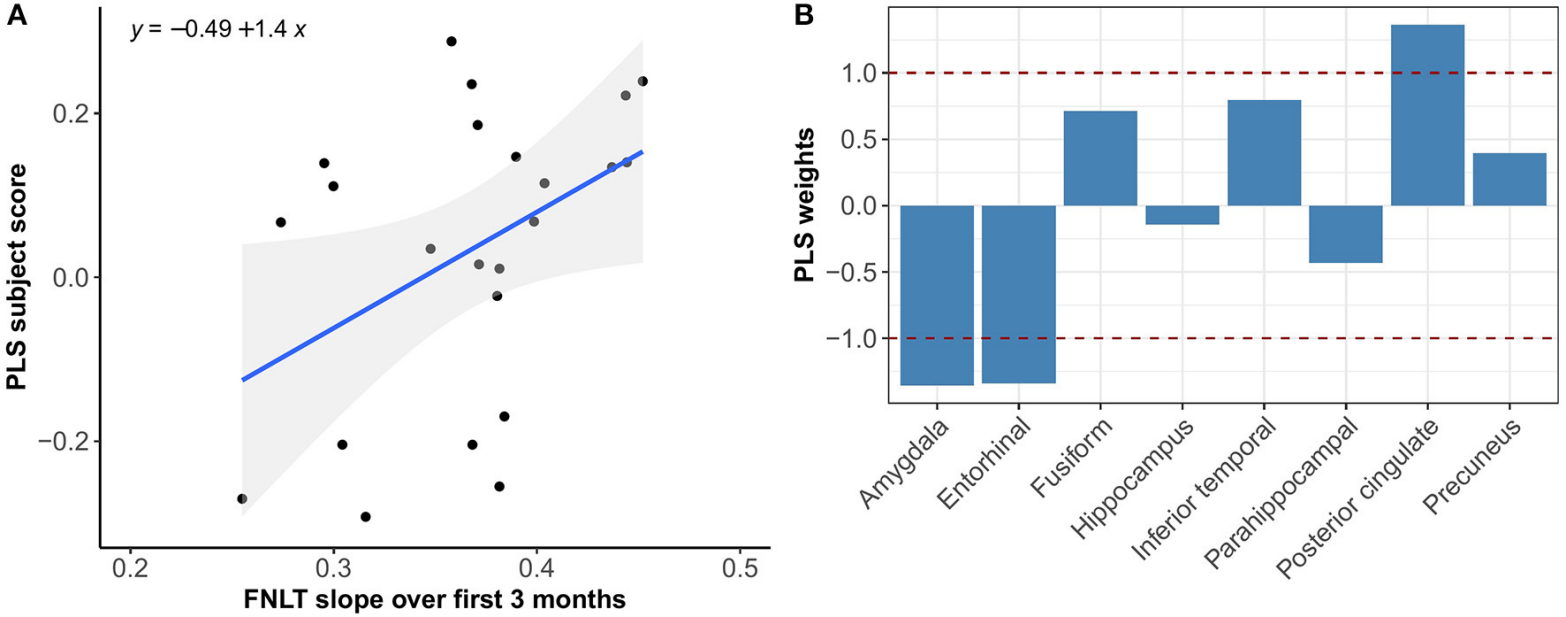
Association between optimal spatial distribution of tau and FNLT slopes over 3 months in the Aβ+ group. Left panel **(A)** shows the correlation between PLS subject scores (expression of spatial patterns of tau distribution) and FNLT slopes; right panel **(B)** presents the spatial patterns of tau distribution of z-transformed PLS weights in each ROI.

Multivariate PLS analyses revealed no spatial patterns of tau distribution that were associated with any of the C3 baseline scores in the overall sample nor in the different Aβ-groups. No spatial patterns of tau distribution were identified that significantly correlated with C3 slopes in the overall sample, but in the Aβ− group we observed a consistent spatial pattern characterized by relatively lower tau uptake in the entorhinal cortex. The expression of this spatial pattern in Aβ− group was significantly associated with higher 3-month slopes on the C3 composite (*p* < 0.001) (Figure 3). The correlation was most pronounced on the BPSO (*p* < 0.001) and OCL accuracy measures (*p* = 0.004) (Figures 4, 5). In the Aβ+ group, we only observed a spatial pattern characterized by relatively lower tau uptake in the amygdala and entorhinal and relatively higher tau uptake in the posterior cingulate. The expression of this pattern was associated with higher 3-month slopes on the FNLT (*p* = 0.003) (Figure 6).

### Diminished Practice Over 3 Months Is Associated With Annual Decline on the PACC5

**Figure 7:**
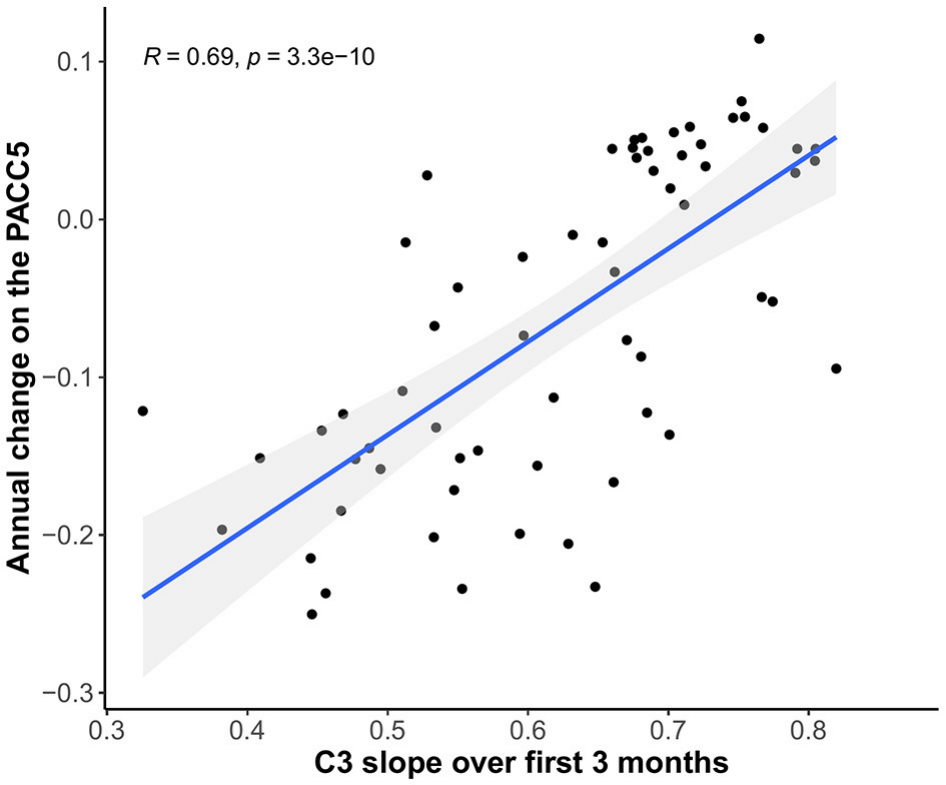
Relationship between C3 slopes over 3 months and annual change scores on the PACC5 (all scores covariate-adjusted).

3-month C3 slopes were positively associated with annual change on the PACC5 (*r* = 0.69, 95% CI [0.55–0.80], *p* < 0.001), indicating that less improvement over 3 months is associated with greater annual PACC5 decline (Figure 7).

**Figure 8:**
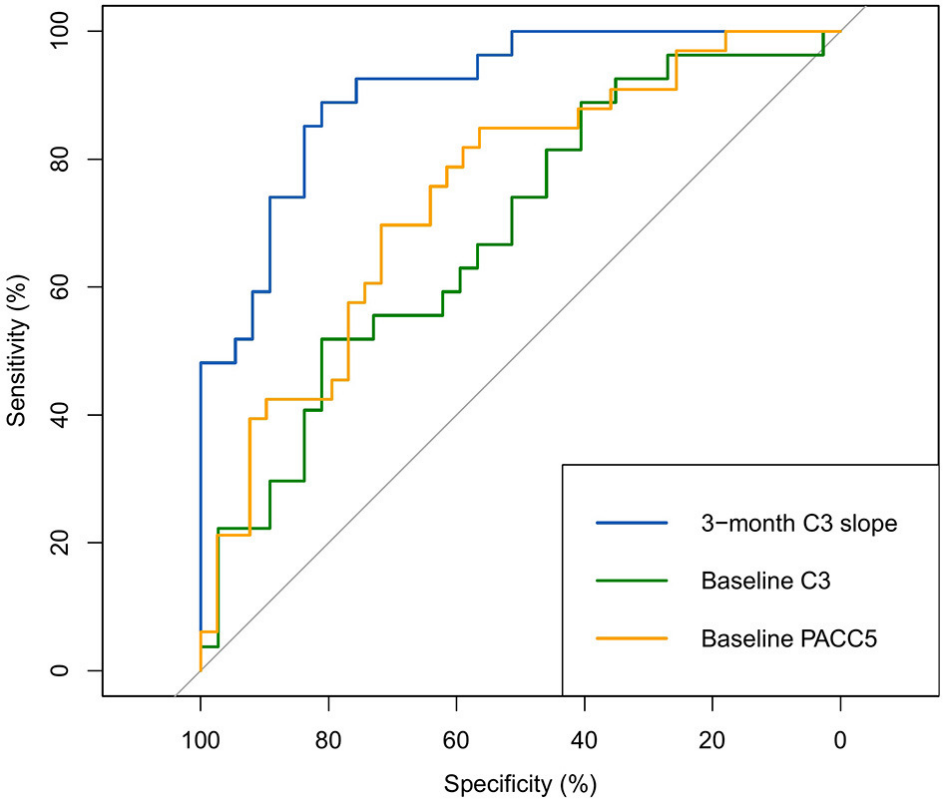
ROC analyses for the identification of >0.10 SD annual decline on the PACC5 (all scores covariate-adjusted).

The ROC analyses presented in Figure 8 show that the 3-month C3 slopes exhibited good discriminative ability to identify individuals who showed >0.10 SD annual decline on the PACC5 (optimal cut-off: 0.7, area under the curve (AUC): 0.91, 95% CI [0.84–0.98], sensitivity = 88.9%, specificity = 81.1%), which was found to perform better than baseline C3 performance (AUC: 0.69, 95% CI [0.55–0.82], *p* < 0.001) and baseline PACC5 performance (AUC: 0.75, 95% CI [0.63–0.86], *p* = 0.02).

When looking at the individual C3 measures, 3-month BPSO and FNLT slopes were more strongly related to annual PACC5 change *(r* = 0.68, 95% CI [0.51–0.79], and *r* = 0.53, 95% CI [0.34–0.68] respectively, both *p* < 0.001), compared to the OCL slopes which only reached trend-level significance (*r* = 0.21, 95% CI [−0.02–0.42], *p* = 0.07). Only BPSO 3-month slopes (optimal cut-off: 0.2, AUC: 0.90, 95% CI [0.83–0.97]) showed significantly better discriminative ability than PACC5 baseline scores (*p* < 0.001), whereas the FNLT 3-month slopes (optimal cut-off: 0.4, AUC: 0.80, 95% CI = [0.70–0.90]) and OCL 3-month slopes (optimal cut-off: 0.1, AUC: 0.60, 95% CI = [0.46–0.73]) did not.

## Discussion

We demonstrated that CU adults improve over monthly computerized cognitive testing, and that, overall, improvement seems most apparent over initial repeated exposures (i.e., over the first four assessments compared to assessments thereafter). However, individuals vary in their magnitude of improvement over 3 months such that attenuated improvement (i.e., diminished practice effect) was associated with greater global amyloid burden and early tau deposition specifically in the entorhinal cortex. Moreover, 3-month C3 slopes were able to detect differences in spatial tau distribution better than C3 baseline scores. Finally, we showed that the magnitude of C3 slopes over 3 months was predictive of cognitive change over 1 year and could provide a valuable marker to identify individuals who will show more than 0.10 SD annual decline on standard paper-pencil cognitive testing.

Improvement over cognitive testing sessions in the absence of an intervention is thought to reflect practice, also referred to as learning or retest effects, which have typically been viewed as source of error or bias in the context of cognitive testing. There is, however, a growing body of literature suggesting that quantifying PE, and particularly lower or reduced PE, could provide a meaningful clinical marker of (early) subtle decrements in learning and memory performance in preclinical stages of AD (Duff et al., 2007; Hassenstab et al., 2015; Jutten et al., 2020a; Lim et al., 2020; Samaroo et al., 2020). One potential explanation for this is that individuals with reduced PE do not optimally benefit from previous exposure to test material, suggesting worse consolidation and retention of recently learned information induced by deficits in the integrity of their learning and memory system. Impairments in learning and retention of new information have been determined to be the earliest and most robust manifestation of AD, which is in line with consistent observations that AD pathology typically manifests earliest in the MTL and specifically the hippocampal and perirhinal regions that play a crucial role in the learning and consolidation system (Reitz et al., 2009). Our finding that lower PE are associated with greater global amyloid burden and tau deposition in the entorhinal cortex contribute to previous work suggesting that we can potentially capture the first, subtle alterations in learning in preclinical AD by capitalizing on the phenomenon of PE (Samaroo et al., 2020).

PLS results complemented the univariate imaging analyses by showing that 3-month C3 slopes detected differences in spatial tau distribution whereas baseline C3 scores did not. In addition, the PLS analysis revealed that the expression of the tau pattern associated with C3 slopes seems different for Aβ− vs. Aβ+ groups. That is, in the Aβ− group we observed a consistent spatial pattern characterized by relatively lower uptake in the entorhinal cortex only, and the expression of this pattern was significantly associated with greater PE over 3 months. In the Aβ+ group we observed a spatial pattern characterized by lower uptake in entorhinal and amygdala and higher uptake in precuneus and posterior cingulate, which was associated with greater PE over 3 months. This difference in spatial patterns could be explained by the fact that Aβ+ group was more progressed in terms of tau pathology than Aβ−, and different relative scales of tau binding in the entorhinal cortex (affected earlier) and posterior regions (affected later) across groups. This is in line with our current understanding that the entorhinal cortex is among the earliest regions of tau accumulation where tau seems to increase with age even before amyloid starts deposing (Maass et al., 2018), whereas other MTL and more posterior regions are affected once amyloid pathology induces the spread of tau into the neocortex (Sanchez et al., 2021). Another interesting difference is that spatial tau distribution in Aβ− was mainly associated with the BPSO and OCL slopes, whereas in the Aβ+ group we only found a significant association with the FNLT slopes. This could be explained by the fact that the nature of the PE observed on the BPSO and OCL (alternate versions) is partly different than the nature of PE on the FNLT (same version), with the latter more heavily relying on remembering the exact test content which may potentially be affected once amyloid pathology is present.

Besides remembering the exact test content, another potential explanation for the occurrence of PE is that increased familiarity with the test-taking in general leads to the development of strategies and/or reduced test anxiety and stress with repeated testing. These task familiarity effects may be due to procedural learning which is an aspect of cognition that remains relatively spared in early stages of AD (Goldberg et al., 2015). Task familiarity has likely played a role in the PE we observed on particularly the OCL and BPSO, since alternate versions were used for those measures and, thus, ruling out the possibility that PE were only caused by the fact that individuals learned/remembered the specific test items. However, the discrepancy between our findings on the BPSO vs. the OCL regarding the magnitude of PE observed over months and the strength of their associations with annual PACC5 decline, also suggest that test familiarity alone seems insufficient to explain differences in PE. In fact, our findings imply that the nature of the task, as well as the retest paradigm (i.e., same vs. alternate versions) may both contribute to the occurrence and magnitude of PE.

When comparing the different tasks and retest paradigms included in the C3, we found that PE were strongest on the FNLT for which the same version was administered at each time-point. However, PE observed on the BPSO, which was administered using an alternate version retest paradigm (A-B-C-D), showed the strongest sensitivity to differences in early entorhinal tau deposition and better predictive ability for annual decline on the PACC5. The BPSO is a measure of pattern separation, an aspect of episodic memory dependent on hippocampal function (Kirwan and Stark, 2007; Yassa and Stark, 2011) whereby information from overlapping experiences is made independent of one another to overcome interference. Our data showed that task performance relying on this process of pattern separation can improve with practice, even though the individual test items at retest are not the same. This suggests that PE observed on the BPSO are not (only) caused by remembering the exact test items, but that practicing strategies to successfully apply pattern separation plays a role as well. The OCL on the other hand, which was also administered using an alternate retest paradigm and initially designed deliberately to mitigate PE, was less sensitive to PE than both the BPSO and FNLT. This could be explained by the fact that the OCL is a “simpler” measure than the BPSO, providing less room for practicing the required learning/memory strategy, which is in line with previous reports that tests with lower cognitive demand show usually lower PE as opposed to tasks with a larger cognitive demand (Beglinger et al., 2005).

Our finding that PE are most strong with initial repeated exposures is in accordance with a previous meta-analysis and several reviews of PE in the context of longitudinal cognitive aging studies (Beglinger et al., 2005; Calamia et al., 2012; Machulda et al., 2013; Jutten et al., 2020a). These studies show consistently that PE at a group level are most apparent between the first- and second-time testing, and that improvement plateaus after 4–5 repeated assessments. However, it is likely that the moment that individuals reach their plateau differs per individual. Therefore, besides quantifying the amount of improvement over a fixed time-interval as we did in the current study, it would be interesting to characterize learning curves at an individual level and investigate whether the number of assessments needed to reach one's personal plateau could provide an early marker of learning deficits in preclinical AD. Additionally, since other studies have suggested that PE can already be detected over days (Lim et al., 2020) or even over repeated assessments within a single day (Darby et al., 2002), it would be interesting to explore the feasibility and predictive ability of defining even more short-term PE (days rather than months) in the context of preclinical AD (Kaye et al., 2021; Papp et al., 2021a).

### Implications

Neuropsychological models have understood the cognitive manifestation of AD in terms of change over years or even decades. However, there is now a developing field that shows how understanding changes in cognition over much shorter periods, such as months, may help inform brain behavior models of the disease, particularly in early or preclinical stages. Our findings provide complementary evidence for the hypothesis that characterizing short-term PE could aid in the detection of individuals at risk for cognitive decline due to AD, above and beyond baseline cognitive scores. This has important implications for clinical trial design and recruitment strategies. First, employing remote, monthly computerized assessments could lead to more rapid recruitment and screening of large samples in a cost-effective manner and maximize sample generalizability by facilitating the inclusion of participants who live in remote locations. Subsequently, characterizing PE over 3 months could advance the more rapid detection of early cognitive change, as well as the identification of those who are at risk for short-term cognitive decline and, thus, may be most likely to benefit from treatment. Ultimately, quantifying PE as a more nuanced way of exploring subtle alterations in cognitive functioning could hopefully increase the rapidity of screening participants and detecting treatment effects in trials that aim slow or halt disease progression in early stages of AD.

Finally, remote cognitive testing may potentially advance the monitoring of (incipient) cognitive impairment in clinical practice. However, not much is known yet about the potential clinical implications of applying a monthly at-home testing paradigm for an individual. For example, the impact of at-home testing on an individual's willingness to have in-clinic follow-up or seek care remains unknown and will thus be an important next step to address in future research.

### Study Limitations

An important limitation of the current study is the fact that our study sample is a highly educated and predominantly White cohort, and thereby it is unknown how generalizable our findings are to other populations. Although adherence in our study was high and missing data due to technical difficulties low, it is important to address that a certain level of digital skills as well as compliance to monthly testing are required to successfully implement these monthly computerized retesting paradigms. The feasibility of at-home computerized testing has previously been demonstrated in HABS and other cohorts (Rentz et al., 2016; Perin et al., 2020), but this should also be determined in more diverse populations and individuals with less digital literacy. Furthermore, it should be noted that we used a study-issued iPad including proprietary CogState software, which may, on the one hand, have contributed to the good adherence, but on the other hand, limits the scalability of the C3.

A general issue with unsupervised cognitive testing is the fact that there is little control over the location, timing of testing, and likelihood of participant distraction, which are all factors likely to interact with task performance and may thereby threaten the internal validity of test scores (Perin et al., 2020). However, a previous study indicated that these factors mainly affect speed of performance rather than accuracy scores (Backx et al., 2020), and since we focused on accuracy measures to detect PE, we do not expect that the uncontrolled environment has biased our findings to a large extent. Furthermore, we applied previously defined cut-offs to ensure the integrity and completion of each individual task, which may also have limited the influence of uncontrolled factors on our results.

A strength of our study is that we complemented univariate imaging analysis with PLS. The main advantage of PLS over univariate analysis is that PLS analysis can examine the relationships between the tau uptake in various regions simultaneously rather than localized tau uptake in each region individually. PLS analysis results are thus expected to be more robust when the input variables are collinear, which is the case with tau uptake in the examined ROI (e.g., uptake in the entorhinal and inferior temporal cortex especially in the Aβ+ group). In addition, PLS analysis may be more robust to noise in the data than univariate analysis. However, it should also be noted that only 8 ROI were included in the PLS analysis. This selection of regions was a-priori defined, based on our initial findings and on what is known about the spread of neocortical tau in cognitively older adults (Johnson et al., 2016; Sanchez et al., 2021). Since our sample consisted mainly of cognitively normal individuals (of which the majority was amyloid-negative), it is expected that there is little or no tau uptake beyond those 8 ROI, and so adding in more regions would likely not benefit our models. An interesting future step would be to investigate the association between PE and tau uptake using voxel-wise analysis. “In addition, it would be worthwhile to use PLS to examine whether regional amyloid accumulation would be associated with the magnitude of PE, especially in amyloid-negative individuals that yet have subthreshold levels of amyloid accumulation (Farrell et al., 2018).

Finally, regarding our investigation of the predictive ability of PE for future cognitive decline, it should be noted that we only had one-year prospective follow-up cognitive testing available. This follow-up duration is particularly short in the context of preclinical AD, which is presumed to be a stage that may last 20 years or longer before the onset of objective cognitive impairment (Sperling et al., 2011). It remains uncertain as to which of our participants would show further cognitive decline and eventually progress to the MCI or dementia stage. Annual data-collection of the HABS cohort is ongoing, which will allow us to address this important question in future research.

## Conclusion

We showed that, while PE commonly occur in CU adults, diminished PE over monthly computerized cognitive testing are associated with greater AD biomarker burden and cognitive decline over one year. Our findings imply that unsupervised computerized testing using monthly retest paradigms can provide rapid detection of diminished PE indicative of future cognitive decline in preclinical AD. This could aid in more rapid detection of individuals at risk for cognitive decline and thereby accelerate clinical trial recruitment and screening as well as the detection of treatment effects.

## Data Availability Statement

The raw data supporting the conclusions of this article will be made available by the authors, without undue reservation.

## Ethics Statement

The studies involving human participants were reviewed and approved by Partners Human Research Committee, which is the Institutional Review Board for the Massachusetts General Hospital and Brigham and Women's Hospital. The patients/participants provided their written informed consent to participate in this study.

## Author Contributions

RJ designed and conceptualized the study, analyzed the data, interpreted the data, and drafted the manuscript. JF, DM, and MP assisted with the data analyses and data interpretation. RA, RB, PM, CS, and MY interpreted the data and performed critical editing of the manuscript. DR, KJ, RS, and KP designed and conceptualized the study, interpreted the data, performed critical editing of the manuscript, and provided study supervision. All authors have read and approved the final version of the manuscript.

## Funding

The Harvard Aging Brain Study was funded by the National Institutes of Health/National Institute on Aging (NIH/NIA) (P01AG036694; Principal Investigators Sperling, Johnson) with additional support from several philanthropic organizations. RJ was supported by a Rubicon grant from the Dutch Research Council (NWO). CS was supported by an R01AG066683-01 award. KP was supported by a K23 award from NIA (1K23AG053422-01).

## Conflict of Interest

RS receives research funding from NIA, Alzheimer's Association, Eli Lilly and Co., and Eisai. She has served as a consultant to AC Immune, Alyn Cytox, Janssen, Neurocentria, Roche, Prothena, and Shionogi but not directly relevant to this study. MY receives research funding from NIA and NIMH, served as a consultant for Pfizer, Eisai, Cognito Therapeutics, Curasen Therapeutics, BPT Pharma and Dart Neuroscience, and is co-founder of Enthorin Therapeutics and Augnition Labs, none of which are directly relevant to this study. PM is employed full time by Cogstate Ltd. The remaining authors declare that the research was conducted in the absence of any commercial or financial relationships that could be construed as a potential conflict of interest.

## Publisher's Note

All claims expressed in this article are solely those of the authors and do not necessarily represent those of their affiliated organizations, or those of the publisher, the editors and the reviewers. Any product that may be evaluated in this article, or claim that may be made by its manufacturer, is not guaranteed or endorsed by the publisher.
