# Supplementary material for: Monthly At-Home Computerized Cognitive Testing to Detect Diminished Practice Effects in Preclinical Alzheimer's Disease
Source: Front Aging Neurosci. 2022 Jan 13;13:800126. doi: 10.3389/fnagi.2021.800126 (PMC8792465; doi:10.3389/fnagi.2021.800126)
Supplement: Supplementary file 1 [file Data_Sheet_1.docx]

Supplementary Material

| **Supplementary Table 1. Variable Definitions and Completion and Performance Checks for the C3** | | | | |
| --- | --- | --- | --- | --- |
| **Cognitive Test** | **Main Outcome** | **Definition** | **Completion Check** | **Performance Check** |
| CBB DET | Speed of performance; mean of the log_10_ transformed RT for correct responses (lmn) | Detection | ≥75% of 35 trials | Accuracy ≥ 70% |
| CBB IDN | Speed of performance; mean of the log_10_ transformed RT for correct responses (lmn) | Identification | ≥75% of 30 trials | Accuracy ≥ 70% |
| CBB OCL | Accuracy of performance; arcsine square root proportion correct (Acc) | One Card Learning | ≥75% of 80 trials | Accuracy ≥ 40% |
| CBB ONB | Speed of performance; mean of the log_10_ transformed RT for correct responses (lmn)  Accuracy of performance; arcsine square root proportion correct (Acc) | One Back | ≥75% of 31 trials | Accuracy ≥ 50% |
| FNAME (FNLT) | FNLT-accuracy of performance (Acc) | FNLT- 1^st^ Letter Name Recall | ≥75% of 12 trials (a.k.a. at least 9 out of 12 on FNMT, FNLT and FSBT) | Accuracy n/a |
| BPSO (BPET, BPXT) | BPXT: The probability of calling a Distractor/Lure stimulus "Similar" minus the probability of calling a New stimulus "Similar". | BPET-object learning phase  BPXT-Pattern Separation Metric | 100% of 40 trials in the learning phase (BPET)  ≥75% of 60 trials in the recall phase (BPXT) | ≥50% Raw Accuracy on the BPXT (given random pressing can result in raw accuracy of 35% or 40%) |

Abbreviations: CBB = Cogstate Brief Battery, FNAME = Face Name Associative Memory Exam, BPS-O = Behavioral Pattern Separation Test-Object; RT = reaction time.

**Supplementary Figure 1.** Overview of completed C3 assessments by study visit (horizontal axis) by study participant (vertical axis). Study participants are ordered and colored by the number of completed visits available (ranging from 2 to 15).

| **Supplementary Table 2. Time estimates extracted from linear mixed models corrected for age, sex, and education, for the FNLT using same versions versus FNLT using alternate versions.** | | | | | | | |
| --- | --- | --- | --- | --- | --- | --- | --- |
|  | **Monthly change over 1 year** | | | **Monthly change over first 3 months** | | | |
|  | **Time** | **95% CI** | **P-Value** | **Time** | **95% CI** | **P-Value** | **MSDR** |
| **FNLT - same versions** | 0.098 | 0.088 – 0.108 | <0.001 | 0.379 | 0.328 – 0.429 | <0.001 | 1.37 |
| **FNLT - alternate versions** | 0.044 | 0.032 – 0.055 | <0.001 | 0.107 | 0.059 – 0.156 | <0.001 | 0.40 |

Abbreviations: FNLT = First Name Letter Test; MSDR = mean to standard deviation ratio.

**Supplementary Results: FNLT with same versus alternate versions**

Improvement over both 12 and 3 months on the FNLT using alternate versions was smaller compared to the FNLT same version (Supplementary Table 2, please note that 95% CIs are not overlapping). 3-month slopes on the FNLT with alternate versions were not associated with one-year change on the PACC5 (*r* = .15, 95% CI [-.08 – 0.37], p=0.2), nor did the FNLT alternate version yield significant correlations with tau distribution as analyzed using PLS. This confirms that applying the alternate version paradigm to the FNAME is less useful to detect differences in PE across individuals.
